# Plasmepsin V, a Secret Weapon Against Malaria

**DOI:** 10.1371/journal.pbio.1001898

**Published:** 2014-07-01

**Authors:** Caitlin Sedwick

**Affiliations:** Freelance Science Writer, San Diego, California, United States of America

*Plasmodium falciparum* and *Plasmodium vivax* are single-celled parasites that, between them, are responsible for the vast majority of malaria cases in humans. Of the two, *P. falciparum* often provokes the most acute symptoms, whereas *P. vivax* is associated with a recurring, chronic version of malarial disease. Both parasites spend a large portion of their life cycle living and replicating within human red blood cells (erythrocytes).

While inside erythrocytes, the parasites express and secrete more than 450 proteins. Each of these proteins has a different function for the parasite, but many of them share a distinctive feature: an amino-terminal motif called the *Plasmodium* EXport ELement (PEXEL). This special sequence of amino acids directs proteins into the export pathway, but it is partially removed while the protein is still within the parasite's endoplasmic reticulum by an enzyme called Plasmepsin V (PMV). On the basis of this prominent function, it seems likely that PMV is important for parasite survival and may therefore be a good target for antimalarial drugs.

Researchers have attempted to test this idea by disrupting the gene that encodes PMV. Standard techniques for gene disruption involve creating a genetic construct containing a gene cassette that confers resistance to a particular drug and inserts itself into the target gene, disrupting the target's coding sequence. Following exposure to the drug, only cells that have integrated the construct (and consequently lost expression of the targeted gene) will survive. But when this approach was attempted with PMV in the malaria parasites *P. falciparum* and *P. berghei*, no drug-resistant parasites were recovered. This suggested that PMV was indeed important to malarial parasites but was not direct proof that it is an essential protein. Brad Sleebs, Justin Boddey, and colleagues describe how they addressed this problem in this month's issue of *PLOS Biology*.

When Sleebs et al. compared *P. falciparum* and *P. vivax* PMV, they found that PMV is highly conserved between the two species. Hypothesizing that PMV is likely essential because of its ability to cleave PEXEL motifs, the authors designed a novel specific inhibitor to disrupt this activity. This inhibitor, dubbed “WEHI-916,” or “916” for short, physically resembles the PEXEL but cannot be cleaved by PMV, so it binds to and blocks the enzyme's active site. Testing showed that 916 inhibited purified *P. falciparum* PMV at a 50% inhibitory concentration of 20 nM, whereas similar but structurally different compounds could not inhibit the enzyme except at much higher concentrations.

**Figure 1:**
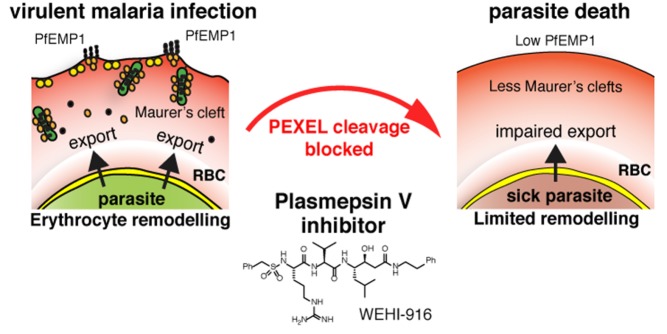
Malaria parasites survive inside red blood cells by exporting proteins that renovate the cell. Inhibition of the export process, by blocking the malarial enzyme, Plasmepsin V, prevents red cell remodeling and kills the parasite. *Image credit: Justin Boddey.*

Encouraged by this result, the authors next tested whether 916 could inhibit PMV when *P. falciparum* is growing in erythrocytes. To do this, they monitored PEXEL cleavage from the PEXEL-containing protein PfEMP3 and found that 916 did inhibit PMV enzymatic activity in live parasites. Further experiments with 916 also allowed new insights into the mechanics of PMV-mediated cleavage. For instance, the researchers were able to determine that PEXEL cleavage takes place almost simultaneously with the protein's synthesis. Prolonged incubation with 916 blocked this process and therefore caused the uncleaved protein to accumulate in the endoplasmic reticulum. Additionally, although 916 had no effect on overall protein translation, it did block the export of another PEXEL-tagged protein called Hyp8. It also prevented export of a key virulence protein called PfEMP1, which lacks a PEXEL tag, but whose export depends upon the activity of PEXEL-tagged proteins (see Figure 1).

Having shown that 916 blocks important PMV activities, Sleebs and colleagues next investigated the compound's impact on parasite viability by treating parasite-infected erythrocytes with the drug. 916 did kill the parasites, and Sleebs et al. found that 916's effective concentration—both for killing and PEXEL cleavage inhibition—could be significantly lowered if PMV protein was first knocked down below wild-type levels. Conversely, higher concentrations of 916 were required to kill parasites and inhibit PEXEL cleavage in parasites overexpressing PMV. Interestingly, PMV knockdown by itself had no effect on either viability or PEXEL cleavage, suggesting that parasites have significant PMV enzymatic capacity to spare.

Another interesting finding in these studies was that 916 could only kill parasites within a certain window of their life cycle. Parasites that have recently invaded an erythrocyte are present in a “ring-stage” form, but they later transition into “trophozoites” and then “schizonts” as they reproduce asexually. Parasites were found to succumb to 916 during the transition from ring-stage to trophozoite. This transition takes place between 20–30 hours after erythrocyte invasion, and 916 must be present throughout this time to kill parasites.

Together, these data show that PMV is indeed required for parasite viability. Although the high concentrations needed for 916 mean this drug cannot be used to treat malaria, future refinements could produce a clinically useful drug. In the meantime, 916 should be a useful tool for probing the biology of malarial parasites.

**Sleebs BE, Lopaticki S, Marapana DS, O'Neill MT, Rajasekaran P, et al. (2014) Inhibition of Plasmepsin V Activity Demonstrates Its Essential Role in Protein Export, PfEMP1 Display, and Survival of Malaria Parasites.** doi:10.1371/journal.pbio.1001897

